# Prevalence and Radiological Characteristics of the Fabella in Turkish Population

**DOI:** 10.7759/cureus.31534

**Published:** 2022-11-15

**Authors:** Hakan Özbay, Hamisi M Mraja, Ata Can, Fahri Erdoğan

**Affiliations:** 1 Orthopedics and Traumatology, Acıbadem Taksim Hospital, Istanbul, TUR; 2 Orthopedics and Traumatology, Istanbul Spine Center, Istanbul Florence Nightingale Hospital, Istanbul, TUR; 3 Orthopedics and Traumatology, Nisantasi Orthopedics Center, Istanbul, TUR

**Keywords:** gastrocnemius muscle, knee mri, sesamoid bone, prevalence, fabella

## Abstract

Background: This study aimed to evaluate and analyze the prevalence and radiological characteristics of the fabella in the Turkish population, detecting differences between genders by examining magnetic resonance imaging (MRI) images of subjects.

Methods: A total number of 504 patients aged >18 years who were admitted to the orthopedics and traumatology clinic between November 2018 and October 2020 were included in this retrospective cross-sectional study. Bilateral MRI images that were taken from each patient were randomly selected. Age, sex, laterality (right or left knee), and size of the fabella were retrieved from institutional database records. P-value<0.05 is considered statistically significant.

Results: A total of 504 patients were included with 213 males and 291 females. The overall prevalence of fabella was 20.63%. The mean length, thickness, and width of the fabella were 6.05 mm, 4.63 mm, and 5.92 mm, respectively, in the overall population. The fabella was significantly wider, thicker, and longer in males compared to females in the Turkish population.

Conclusion: This study revealed similar prevalence rates of the fabella in the Turkish population with Caucasian populations and similar size of the fabella in the Asian population. When different prevalence rates and sizes of the fabella among different ethnic populations are considered, it is critical to understand the prevalence or radiological features of the fabella in Turkish subjects to avoid misinterpretation of fabella diseases.

## Introduction

The fabella, also known as "little bean," is a fibrocartilaginous or bony sesamoid bone that is frequently found in the tendinous portion of the lateral head of the gastrocnemius muscle (LG). The fabella frequently articulates with lateral femoral condyle and is rarely seen on the medial side. An ossified fabella is usually seen at the age of 12 years or older in 10-30% of the population [1].

The fabella is often seen bilateral as a small, round ossification. Its main function is thought to stabilize lateral femoral condyle and fabella complex, which consists of plantaris and gastrocnemius muscles (arcuate, fabellofibular, and oblique popliteal ligaments) [2]. Fractures, dislocations, fabella pain syndrome, or common peroneal nerve palsy are some of the clinical conditions related to traumatic or non-traumatic scenarios [3-6].

The fabella is assumed to be more common in Asians, equally common between genders, and more common in the older population; and it is thought that the fabella is ~3.5 times more common today than 100 years ago. And also the presence or absence of the fabella is considered a normal variant. Some genetic and environmental causes have been hypothesized for this variation in frequency or size [7].

Given its effects on some clinical conditions and variations in prevalence or morphologic characteristics as mentioned above, it is important to understand the change in prevalence rates between ethnic groups or the size of the fabella. This study aimed to investigate the prevalence and radiological characteristics of the fabella in the Turkish population, analyzing differences between genders by examining magnetic resonance imaging (MRI) images of subjects.

## Materials and methods

Study design and patients

A retrospective cross-sectional study was designed with a total number of 504 (291 female and 213 male, aged from 19 to 71 years, 1,008 knee MRI images) patients who were admitted to Agri Training and Research Hospital Clinic of Orthopedics and Traumatology for any complaints between November 2018 and October 2020. Patients aged >18 years old who presented to our clinic with any complaints and were examined by bilateral knee MRI were included in this study. Patients who had a history of knee surgery, patients with advanced osteoarthritis where discrimination of osteophytes and fabella is difficult and MRI images with a low-quality image to measure study parameters were excluded. Patient data were collected from the institutional hospital database. Bilateral MRI images that were taken from each patient were randomly selected and evaluated. Age, sex, laterality (right or left knee), and symmetry pattern of the fabellae were retrieved from institutional database records. Also, the maximum length, thickness, and width of the fabellae and the distance of the fabellae to the insertion point of lateral or medial gastrocnemius were measured using MRI views and recorded for statistical analysis. Our research was conducted in accordance with the principles set forth in the Helsinki Declaration, 2008. Ethical approval for the study was obtained from the Clinical Research Ethics Committee of our hospital (date: November 23, 2020; number: 18).

Radiographic assessment

**Figure 1:**
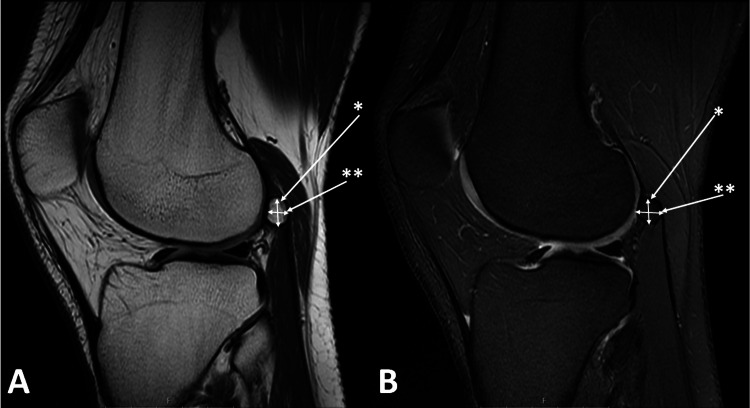
Sagittal proton density-weighted (A) and proton density fat-saturated (B) MRI views demonstrating the measurement of fabella’s length (*) and thickness (**).

**Figure 2:**
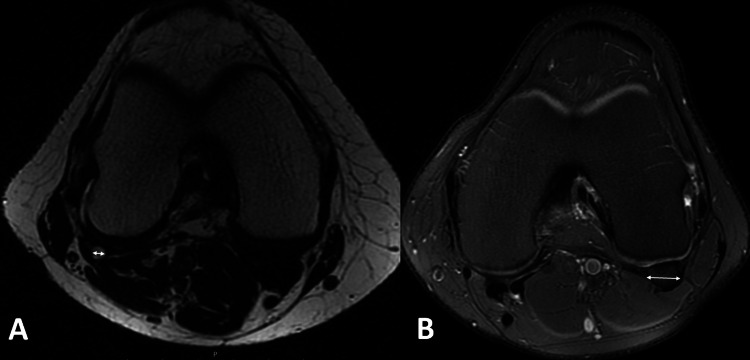
T2 (A) and axial proton density fat-saturated (B) MRI views demonstrating the measurement of fabella’s width using the double arrow.

Sagittal and axial views of each MRI image were evaluated. The maximum length and maximum thickness of the fabella were measured by using sagittal MRI views (Figures 1A, 1B), and axial MRI views were used to measure the maximum width of the fabella in patients who had a fabella (Figures 2A, 2B).

**Figure 3:**
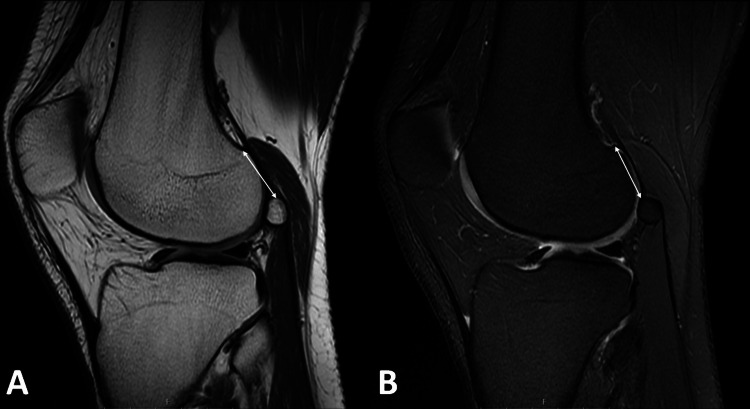
Sagittal proton density-weighted (A) and proton density fat-saturated (B) MRI views demonstrating the measurement of the distance between fabella to the insertion point of the lateral head of the gastrocnemius muscle (LG) using the double arrow.

Also, the distance of fabella to the insertion point of the LG on the femur was measured by using sagittal MRI views and documented (Figures 3A, 3B). Furthermore, medial side fabella measurements were performed according to the medial gastrocnemius. All MRI images were reviewed by two orthopedic surgeons independently. They determined the locations of the fabellae in sagittal and axial MRI views and measured the length, width, thickness of the fabellae, and the distance of the fabellae to the insertion point of lateral or medial gastrocnemius on the femur. The final decision was reached by consensus after the determination of uncertain cases. All demographical and radiological data were collected by the researcher to be analyzed statistically.

Statistical analysis

A power analysis was performed using G*Power, version 3.1.9.4 (University of Kiel, Kiel, Germany) to calculate the sample size. Based on a power of 0.80 with α=0.05 to detect between-group differences, the sample size was identified to be large enough [8]. Randomization was achieved by using a simple random sampling method. Number Cruncher Statistical System (NCSS, Kaysville, Utah, 2007) program was used for statistical analysis. Descriptive statistical methods (mean, standard deviation, median, frequency, ratio, minimum, maximum) were used while evaluating the demographical features of the patients, and some radiological parameters (the prevalence, side, and laterality) of the fabellae. Pearson chi-square test was used to analyze the differences between fabellae at their location and side and genders. P<0.05 is considered statistically significant.

## Results

A total of 504 patients were included of which 213 (42.26%) were males and 291 (57.73%) were females. The mean age of patients was 39.06±11.9 (mean±standard deviation) years, ranging from 19 to 71 years. The overall prevalence of fabella was 20.63% (N=104). A total of 63.46% (N=66) of these patients had the fabella bilaterally and 36.53% (N=38) of these patients had the fabella unilaterally. Of these patients who had fabella unilaterally, 29 (76.31%) patients had fabella on the right knee and nine (23.68%) patients on the left knee. All the fabellae were bony and no cartilaginous fabella was detected in this study.

The overall prevalence of fabella was 21.21% (N=45) in males and 20.27% (N=59) in females. There was no significant difference between gender and prevalence of fabella (p>0.05). All the fabellae were located within the lateral head of the gastrocnemius, except three patients who had fabella in the medial head of the gastrocnemius, two in the right knee and one in the left knee.

The overall mean length of the fabella was 6.05±1.4 (3.7-9) mm, with 6.84 mm in males and 5.26 mm in females. The mean thickness of the fabella in the overall population was 4.63±1.09 (3.3-7.2) mm. The mean thickness of the fabella was 4.84 mm in males and 4.42 mm in females. The overall mean width of the fabella was 5.92±1.2 (4.3-8.6) mm, with 6.35 mm in males and 5.49 mm in females. The fabella was significantly wider, thicker, and longer in males compared to females in the Turkish population (p<0.01) (Table 1).

**Table 1:** Comparison of the radiological parameters of the fabellae between genders. LG: lateral head of the gastrocnemius; SD: standard deviation *Pearson chi-square test; p<0.05 was considered statistically significant.

Variables		Female	Male	Total	p-Value
		Mean±SD (range)	Mean±SD (range)	Mean±SD (range)	
Parameter	Length of the fabella (mm)	5.26±1.22 (3.68-6.75)	6.84±1.66 (4.97-8.99)	6.05±1.4 (3.68-8.99)	<0.01*
	Thickness of the fabella (mm)	4.42±0.9 (3.32-6.02)	4.84±1.24 (2.9-7.8)	4.63±1.09 (2.9-7.8)	<0.01*
	Width of the fabella (mm)	5.49±1.12 (3.47-7.61)	6.35±1.43 (3.8-9.12)	5.92±1.2 (3.47-9.12)	<0.01*
	Distance of the fabella to the insertion point of the LG on the femur (mm)	28.31±1.86 (25.4-33.1)	35.48±2.32 (27.1-43.6)	31.52±1.94 (25.4-43.6)	<0.01*

The average distance of the fabella to the insertion of LG on the femur in the overall population was 31.52±1.94 mm. It was 30.40 mm in the right knees and 32.64 mm in the left knees of patients. There was no significant difference between laterality and distance of fabella to the insertion point of LG on the femur (p>0.05). Females had the fabella closer to the insertion point of LG onto the femur at 28.31 mm than males at 35.48 mm (p<0.01) (Table 1).

## Discussion

The fabella is considered to be of minor clinical significance among clinicians because of its rarity and benign structure. However, there are some clinical conditions or diseases related to fabella and it could cause a delay in diagnosis without comprehensive knowledge about this sesamoid bone. And also it could cause unnecessary surgical interventions or medications without the ability to distinguish them from loose bodies or osteophytes [9]. When differences in prevalence rates and morphological or radiological characteristics among ethnic populations were considered, it would be appropriate to describe the prevalence and radiological features of this sesamoid bone in the Turkish population, as intended in this study.

The prevalence of fabellae is different among ethnic populations. Reported prevalence changes between 3.1% and 31.3% in the Caucasian population and 30.6% and 92% in the Asian population. There is no accepted genetic reason for these variations. However, they hypothesized that persistent pressure on the fabella against the lateral femoral condyle could promote the development and ossification of the sesamoid bone. So, it explains the higher prevalence of the fabella in the Asian population who prefer kneeling, squatting, etc. [10-13]. Chew et al. investigated the prevalence of fabella in the Asian population, evaluating arthroscopic examination of 80 patients and they found the prevalence of fabella as 31.25% [14]. Also, they found no statistically significant difference between the incidence of the fabella and gender. In this study, they stated that the mean length, thickness, and width were 7.06 mm, 4.89 mm, and 6.12 mm, respectively. In my study, the mean length, thickness, and width of the fabella were 6.05 mm, 4.63 mm, and 5.92 mm, respectively in the overall population. However, the fabella was wider, thicker, and longer in males compared to females. Although the dimensional parameters of the fabella in the overall population were similar to the Asian population; the difference between genders and dimensional parameters was not parallel with studies in the Asian population. 

Egerci et al. found in their studies in which they evaluated 500 patients, 250 male and 250 female, with plain radiographs that the overall prevalence of fabella was 22.8% [15]. The fabella was present bilaterally in 15.2% of the population in their study. Also, they stated that the prevalence of the fabella was similar between genders. Findings in this study that evaluate the overall prevalence, laterality, and distribution between genders were consistent with the literature. The overall prevalence was 20.63% and 13.09% of the population had the fabella in both knees. These results were similar to the Caucasian populations. Also, there was no statistically significant difference between genders and prevalence of the fabella in this study.

The prevalence of the morphological characteristics of fabella can be studied using observational methods like plain radiography, computed tomography, MRI, or cadaver investigations. MRI images were preferred for this study because they allowed the detection of any cartilaginous fabella whenever present. There was no cartilaginous fabella in this study, all the fabellae were bony. The reason for this finding is presumably that patients <19 years of age were excluded from this study. And also, it would be appropriate to use sagittal cuts of MRI to measure the distance of the fabella to the insertion point of LG onto the femur. In this study, the average distance of the fabella to the insertion of LG on the femur in the overall population was 31.52 mm which is a consistent result with the study in the Asian population [14]. The fabellae of females were significantly closer to the insertion point of LG on the femur than males. Also, this result was not consistent with the existing literature.

The fabella is mostly found in the tendinous portion of the lateral head of the gastrocnemius muscle. It is rarely found in the medial head of the gastrocnemius muscle. In a cadaveric study by Zeng et al. on 61 specimens, they found six cases having a fabella in the medial head [16]. In this study, all the fabellae except three cases were in the tendinous portion of LG. Two of these three cases were in the right knee and one in the left knee. These results were also consistent with the literature. It is not evaluated in this study whether the location of the fabella on the medial or lateral side causes disease or not, due to its retrospective design. In our knowledge, this study is the first and only study in the literature about the prevalence, length, thickness, width, location, and symmetry pattern of the fabella evaluated using MRI on Turkish subjects.

This study also has some limitations. It is a registry-based study designed in one hospital in a local geographical area. So, it could not reflect the exact prevalence or radiological characteristics of the fabella in the entire population. Further multicenter studies are needed with a larger population to give more precise results. Because of its retrospective design, diseases or symptoms of patients and their relation to the location or size of the fabella could not be analyzed. Another limitation is that patients <19 years of age were not included in this study and so the prevalence of cartilaginous fabella could not be detected. Also, this study is designed to measure dimensions on MRI images and it could not be as precise as cadaveric studies. But it provided us to study a higher number of cases (1,008 MRI images).

## Conclusions

Similar prevalence rates of the fabella with Caucasian populations and similar size of the fabella with Asian populations were detected in this study. The fabella was thicker, wider, and longer in males; and also females had fabellae closer to the insertion point of LG. When misinterpretation or underdiagnosis of fabella diseases is considered, it is critical to understand the prevalence or radiological features of the fabella.
